# Relationship between abstract thinking and eye gaze pattern in patients with schizophrenia

**DOI:** 10.1186/1744-9081-10-13

**Published:** 2014-04-16

**Authors:** Jooyoung Oh, Ji-Won Chun, Jung Suk Lee, Jae-Jin Kim

**Affiliations:** 1 Department of Psychiatry, Yonsei University College of Medicine Gangnam Severance Hospital, 211 Eonjuro, Gangnam-gu, Seoul 135-720, Korea; 2 Institute of Behavioral Science in Medicine, Yonsei University College of Medicine, Seoul, Korea; 3 Department of Psychiatry, Bundang Jesaeng Hospital, Seongnam, Korea

**Keywords:** Schizophrenia, Abstract thinking, Theme identification, Eye gaze

## Abstract

**Background:**

Effective integration of visual information is necessary to utilize abstract thinking, but patients with schizophrenia have slow eye movement and usually explore limited visual information. This study examines the relationship between abstract thinking ability and the pattern of eye gaze in patients with schizophrenia using a novel theme identification task.

**Methods:**

Twenty patients with schizophrenia and 22 healthy controls completed the theme identification task, in which subjects selected which word, out of a set of provided words, best described the theme of a picture. Eye gaze while performing the task was recorded by the eye tracker.

**Results:**

Patients exhibited a significantly lower correct rate for theme identification and lesser fixation and saccade counts than controls. The correct rate was significantly correlated with the fixation count in patients, but not in controls.

**Conclusions:**

Patients with schizophrenia showed impaired abstract thinking and decreased quality of gaze, which were positively associated with each other. Theme identification and eye gaze appear to be useful as tools for the objective measurement of abstract thinking in patients with schizophrenia.

## Background

Abstract thinking is defined as the ability to think beyond the immediate, specific stimulus situation and to think about situations in general, symbolic modes [1]. Abstract thinking is the opposite of concrete thinking, which is thinking of objects or ideas as specific items. Deficits in abstract thinking are prominent in patients with schizophrenia, particularly in chronic patients and patients with severe symptoms [1]. Deficits in abstract thinking can cause patients with schizophrenia to run into substantial difficulties in social interactions during daily life and work life [2,3].

Many studies have investigated abstract thinking in schizophrenia [1,3-7], but most have used neuropsychological tests, including the Wisconsin card-sorting test. This test evaluates abstract thinking ability, such as rule-based categorization [8], and is a measure for other aspects of the executive functions, including cognitive flexibility [9,10]. Some studies focused on abstract thinking using the Gorham proverb test or the Benjamin proverb test [1,4,5], but results from these tests can be influenced by education level and general frontal lobe functions such as working memory [11].

Measurement of eye gaze can be a powerful way to analyze the processing of visual information [12]. Eye gaze has an important role in cognition through attention, reading, and scene perception [13]. A “top-down” process makes an important contribution to the control of eye movement in that the cognitive and affective status of a person is significant in eye gaze [14]. Previous studies on eye gaze have demonstrated that patients with schizophrenia clearly show atypical eye gaze patterns or scan paths [15,16]. Restricted scanning in patients with schizophrenia has a close relationship with negative symptoms, including avolition and affective blunting, deficits in executive functions, and problems with social interaction [16,17].

It has been reported that healthy individuals direct their gaze according to their purpose and focus their gaze on the principal component, whereas patients with schizophrenia have slow eye movement and usually explore limited visual information [18]. These abnormal gaze patterns and limited visual searching may make it difficult for them to obtain significant information about objects and then integrate the information. In addition, effective integration of visual information is necessary to utilize abstract thinking [3]. In the draw-a-person technique [19], for example, abstract thinking was evaluated by checking for integrative information in the context of the proportion of each part of the whole person, integrity of the figure, and consistency of lines throughout the figure [20]. Taken together, this evidence suggests that abnormal eye gaze is related to impairment in abstract thinking. However, no one has yet studied the association between visual searching and abstract thinking in patients with schizophrenia.

The aim of this study was to elucidate the nature of the relationship between abstract thinking and visual searching in schizophrenia. To investigate our hypothesis that impairment in abstract thinking is associated with severity of abnormal eye gaze in patients with schizophrenia, we created a theme identification task, in which subjects judged a theme of presented pictures while their visual scan paths were concurrently monitored. We selected theme identification in this task in order to exclusively evaluate abstract thinking ability.

## Method

### Participants

Twenty patients with schizophrenia (11 males) and 22 healthy controls (12 males) participated in this study. All patients were recruited from outpatient clinic of Gangnam Severance Hospital, and healthy controls were recruited using open advertisement on the Internet. The exclusive diagnoses of schizophrenia in the patient group and the exclusions of psychiatric disorders in the control group were made based on the Structural and Clinical Interview for DSM-IV (SCID-IV) [21]. Exclusion criteria included the presence of mental retardation, significant neurological or medical illness, and severe ophthalmological diseases. All patients were taking atypical antipsychotics, and the mean chlorpromazine equivalent dose was 513.9 ± 590.8 mg. This study was approved by the institutional review board of Yonsei University Severance Hospital. All participants provided voluntary written, informed consent before the study began.

### Behavioral tasks

**Figure 1:**
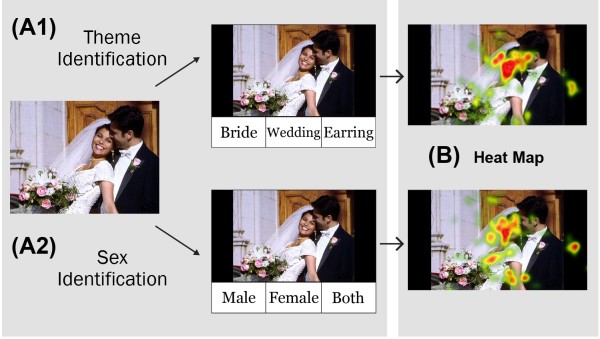
**An example of the experimental tasks and analysis.** The experiment consisted of theme or sex identification for consecutively presented pictures. During theme identification **(A1)**, participants were asked to select the theme word. In this example, “Wedding” was the theme word, “Bride” was the related word, and “Earring” was the unrelated word. During sex identification **(A2)**, participants were asked to select the sex of the people in the picture. In the analysis **(B)**, a heat map was automatically revealed by the eye tracker, and the area of interest (AOI) was defined as the area that healthy controls had viewed for more than 50 ms (colored dark red on the heat map).

We developed a theme identification task to evaluate participants’ abstract thinking ability, and the stimuli used consisted of 20 emotional (10 positive and 10 negative) pictures from the International Affective Picture System (IAPS) [22]. Three words were presented in the bottom of the pictures, and participants were asked to indicate the main theme of the picture by selecting one of these words. The three words were chosen to indicate a main theme word (“theme word”), to be related to but more concrete than the theme word (“related word”), and to be present in the picture but have no direct relationship to the main theme (“unrelated word”). For example, as presented in Figure 1, *wedding* was the theme word (the correct answer), *bride* was the related word, and *earring* was the unrelated word. In order to determine the stimuli to be used in this study, 15 normal participants (8 males) participated in a pilot theme identification task, and only stimuli with correct rates of more than 60% were selected. There were 20 task trials, and each individual trial consisted of two phases that lasted for a total of 6 seconds. In the first 3-second phase, a stimulus picture was presented without words, and participants were asked to explore the picture and think about what the theme was (searching phase). In the next 3-second phase, three words were presented, and participants were instructed to select one of them (reaction phase).

As a control task for theme identification, a sex identification task was employed to evaluate participants’ ability to think concretely. The same 20 pictures presented in the theme identification task were used for the sex identification task, and participants were asked to decide whether the picture included only a male, only a female, or both. The sex identification task also consisted of the searching phase for 3 seconds and the subsequent reaction phase for 3 seconds.

The correct rates and response times for the two tasks were examined using statistical analysis, as described below. In the theme identification task, the selection rates of the related and unrelated words as wrong answers were also calculated.

### Eye movement recordings

Eye movement recording was performed using the SensoMotoric Instrument (SMI, Boston, USA) eye movement monitoring system, which included the Remote Eye Tracking Device 5 (RED5, infrared eye camera), Experiment Center 3.0 (used for stimuli presentation), and SMI iView (eye tracking software). The gaze data were analyzed using SMI BeGaze. In order to maintain a stable head position and reliable detection of gaze, the jaws of participants were fixed with a special device, and the eye position was always measured at a distance of approximately 70 cm from the computer monitor. Before the main experiment, participants were instructed to fix their gaze at several circles to calibrate and validate their eye movements. The sampling rate of eye gaze was 120 Hz; therefore, the maximum number of samples during the searching phase was 360. The numbers of samples, fixations, and saccades were measured. A fixation was counted when participants gazed at the monitor within an area of 100 pixels for more than 80 ms. A saccade was counted between fixation and fixation, between blink and blink, or between fixation and blink.

To compare between patients and controls with respect to the eye gaze patterns that fell within the core area gazed at by the controls, we set up the “area of interest” (AOI), which was defined as the area that controls looked at for more than 50 ms. The rate of fixation in the AOI during the searching phase was calculated for each subject and stimulus.

### Clinical and cognitive measures

Another measure of abstract thinking ability was the Similarities subtest (range: 0–28) in the Korean version of the Wechsler Adult Intelligence Scale [23]. Intelligence was measured using the Raven’s standard progressive matrices (RPM) [24]. Anhedonia was measured using the Physical and Social Anhedonia Scale [25]. To identify clinical symptoms and antipsychotics-induced Parkinsonian symptoms of patients, the Positive and Negative Syndrome Scale (PANSS) [26] and Simpson-Angus Scale (SAS) [27] were employed.

### Statistical analysis

Demographic and clinical data and rates of correct responses were compared between the two groups using independent-sample *t*-tests, except for sex, which was analyzed using the Chi-square test. Gaze data of sample, fixation, and saccade counts were used as the dependent variables in repeated measures analysis of variance (ANOVA), with the group as a between-subjects factor and the task type (theme identification versus sex identification) as a within-subjects factor. In each group, correlation analyses were performed on clinical and demographic data, the rates of correct responses, and eye gaze data. Linear regression analysis was used to determine the interaction effects of group and quality of gaze with respect to the rate of correct responses for each task.

## Results

### Demographic and clinical characteristics

The demographic and clinical data are listed in Table 1. There were no significant differences between patients and controls for age and sex, but education years were significantly fewer (*t* = 2.46, *df* = 30.5, *d* = 0.77, *p* = 0.02) in patients. The between-group difference in raw scores of the RPM was not statistically significant (*t* = 1.75, *df* = 28.9, *d* = 0.55, *p* = 0.091), but raw scores of the Similarities subtest were significantly lower in patients than in controls (*t* = 3.12, *df* = 40, *d* = 0.96, *p* = 0.003). Scores of the Physical and Social Anhedonia Scale were significantly higher in patients (physical, *t* = 2.12, *df* = 40, *d* = 0.67, *p* = 0.04; social, *t* = 3.09, *df* = 40, *d* = 0.95, *p* = 0.004).

**Table 1:** Clinical characteristics of the subjects

	**Control (N = 22)**	**Schizophrenia (N = 20)**	p-value*
Male/Female	12/10	11/9	0.610
Age (years)	30.2 ± 11.7	31.4 ± 9.3	0.720
Education (years)	15.7 ± 1.4	14.3 ± 2.3	0.020
RPM	50.6 ± 8.3	43.9 ± 15.2	0.091
Similarity	18.7 ± 3.9	14.7 ± 4.5	0.003
Physical Anhedonia	12.2 ± 8.1	18.4 ± 10.1	0.035
Social Anhedonia	10.1 ± 6.8	16.6 ± 6.7	0.004
Simpson-Angus Scale		1.0 ± 1.4	
PANSS_positive		13.5 ± 5.3	
PANSS_negative		17.3 ± 5.8	
PANSS_general		33.4 ± 10.1	
Duration of illness (years)		4.5 ± 5.8	
Chlorpromazine equivalent dose (mg)		513.9 ± 590.8	

*by chi-square for categorical variable and independent sample *t*-test for continuous variable. *RPM*: raw score of Raven’s standard Progressive Matrices, Similarity: Score of the Similarity test, *PANSS*: Positive and Negative Syndrome Scale.

### Tasks performance

**Figure 2:**
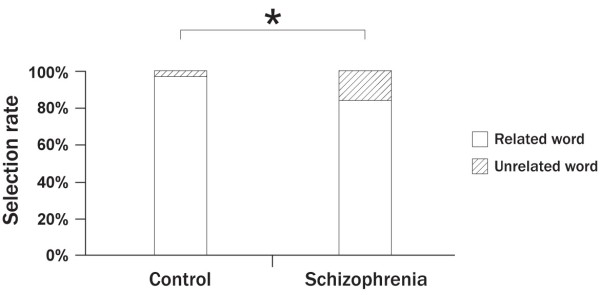
Comparison of the patient and control groups for the selection rates of related versus unrelated words in the trials with wrong selections in the theme identification task (* P < 0.05).

As shown in Table 2, the rates of correct theme identification were significantly lower in patients (81.0 ± 17.0%) than controls (91.1 ± 9.9%) (*t* = 2.39, *df* = 40, *d* = 0.73, *p* = 0.02). This group difference was significant during the negative emotional condition (*t* = 2.30, *df* = 30.7, *d* = 0.72, *p* = 0.03), but not during the positive condition. However, the rates of correct sex identification showed no difference between patients (90.1 ± 9.7%) and controls (90.7 ± 8.6%). In addition, as shown in Figure 2, there was a significant difference between the two groups in the selection rate of related words among incorrect responses during the theme identification task (control, 97.7 ± 6.8%; schizophrenia, 84.1 ± 21.0%; *t* = 2.44, *df* = 32, *d* = 0.87, *p* = 0.02). In other words, the proportion of unrelated words among the wrong answers was significantly higher in patients with schizophrenia than controls. There was no group difference in the response time for either the theme or the sex identification tasks.

**Table 2:** Behavioral responses in the theme and sex identification tasks

	**Control (N = 22)**	**Schizophrenia (N = 20)**	p-value*
**Theme identification**			
Correct rate (%)			
Positive	91.7 ± 10.5	84.9 ± 19.9	0.167
Negative	89.9 ± 12.3	77.8 ± 20.4	0.029
Overall	91.1 ± 9.9	81.0 ± 17.0	0.022
Response time (msec)			
Positive	1629.3 ± 254.9	1534.0 ± 391.3	0.351
Negative	1704.5 ± 263.7	1682.5 ± 405.6	0.835
Overall	1666.9 ± 242.6	1608.3 ± 377.3	0.549
**Sex identification**			
Correct rate (%)			
Positive	88.2 ± 11.0	87.2 ± 12.6	0.794
Negative	93.2 ± 8.4	94.0 ± 8.5	0.747
Overall	90.7 ± 8.6	90.1 ± 9.7	0.846
Response time (msec)			
Positive	1060.4 ± 274.6	1122.8 ± 526.7	0.639
Negative	1030.5 ± 477.3	1032.2 ± 528.6	0.991
Overall	1045.4 ± 339.1	1077.5 ± 518.6	0.812

*by independent sample *t*-test.

### Eye gaze results

For the fixation and saccade counts, there was no significant main effect of the task type and no significant group × task-type interaction. However, there were significant main effects of group on fixation [*F* (1,40) = 6.78, *partial η*^*2*^ = 0.145, *p* = 0.01] and saccade [*F* (1,40) = 6.54, *partial η*^*2*^ = 0.141, *p* = 0.01] (Table 3). Patients showed lower fixation and saccade counts than controls during both tasks [theme (fixation: *t* = 2.24, *df* = 38.3, *d* = 0.69, *p* = 0.031; saccade: *t* = 2.12, *df* = 37.2, *d* = 0.66, *p* = 0.041), sex (fixation: *t* = 2.84, *df* = 40, *d* = 0.88, *p* = 0.007; saccade: *t* = 2.86, *df* = 40, *d* = 0.87, *p* = 0.007)]. For the fixation rate in the AOI and the sample count, there were no significant main effects of the group or task type and no significant group × task-type interaction.

**Table 3:** Eye gaze results during the theme and sex identification tasks

	**Control (N = 22)**	**Schizophrenia (N = 20)**	p-value*
**General eye gaze**			
Abstract or Theme			
Sample count	338.7 ± 8.6	323.7 ± 41.2	0.102
Fixation count	17.4 ± 7.3	12.1 ± 8.1	0.031
Saccade count	19.1 ± 6.9	14.1 ± 8.2	0.041
Concrete or Sex			
Sample count	335.7 ± 12.6	319.7 ± 53.9	0.184
Fixation count	17.2 ± 7.3	10.4 ± 8.1	0.007
Saccade count	18.5 ± 6.1	12.2 ± 8.2	0.007
**AOI gaze**			
Abstract or Theme			
AOI fixation/Total fixation (%)	7.5 ± 6.7	5.5 ± 5.2	0.300
Concrete or Sex			
AOI fixation/Total fixation (%)	7.3 ± 5.7	7.4 ± 6.5	0.955

*by repeated measures analysis of variance and post hoc *t*-test.

*AOI*, area of interest.

### Correlations

The rates of correct theme identification were significantly correlated with the scores of the Similarities subtest (*r* = 0.55, *p* = 0.01) and scores on the Social Anhedonia Scale (*r* = −0.58, *p* = 0.01) in patients, but not in controls. There was no other significant correlation between task performance and clinical variables including years of education and general intelligence in either group.

Fixation count (theme: *r* = 0.48, *p* = 0.03; sex: *r* = 0.57, *p* = 0.01) and saccade count (theme: *r* = 0.59, *p* = 0.01; sex: *r* = 0.65, *p* = 0.002) in both tasks were correlated with the intelligence score in patients, while only fixation count (theme: *r* = 0.47, *p* = 0.03, sex: *r* = 0.44, *p* = 0.04) in both tasks was correlated with the intelligence score in controls. Eye gaze results in both tasks did not show any significant correlation with scores of the SAS and doses of antipsychotics in patients.

**Figure 3:**
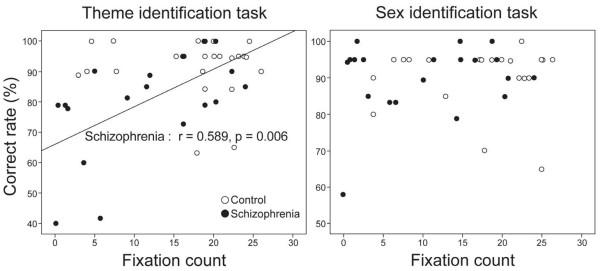
**Relationship between the eye fixation counts and rates of correct selections in each group.** A significant correlation was found only during the theme identification task in the patient group.

Linear regression analysis showed a significant interaction effect (*p* = 0.009) between group and fixation count with respect to the rate of correct theme identification (equation for controls: correct rate = 93.463 - 0.135 × fixation count; equation for patients: correct rate = 65.978 + 1.236 × fixation count), but no significant interaction effect was found with respect to sex identification. There was a significant correlation between the rates of correct theme identification and fixation counts in patients (*r* = 0.59, *p* = 0.006), but not in controls. There was also no significant correlation between the rate of correct sex identification and fixation in controls or patients (Figure 3).

## Discussion

In this study, we developed a new theme identification task to exclusively assess abstract thinking ability. As expected, patients scored worse for theme identification and for reasoning similarities than controls, and they showed a significant correlation between scores of the Similarities subtest and rates of correct theme identification. These results are consistent with previous findings of deficits in abstract thinking in schizophrenia [1,3-7], suggesting that our task may be an effective alternative measurement. Another major result of this study is that patients selected unrelated words more often as wrong answers during theme identification than controls. Because the unrelated words were the most concrete words for the picture, these results suggest that patients tend to think more concretely than controls. Given that the selection rate of related words as wrong answers could detect subtle differences in abstract thinking, our task may be an alternative or supplementary tool for the evaluation of abstract thinking.

In contrast with earlier findings of abnormal results on the proverb test only in older people [11,28], our younger patients showed abnormalities in identifying the theme, suggesting that detection of deficits in abstract thinking at a younger age may be an advantage of our task. Another advantage of our task is to provide a measure of performance that is unaffected by education or general knowledge unlike the proverb test, and to reflect judgments in some situations similar to real life. However, given that patients with severe symptoms exhibited increased impairment in abstract thinking in a previous report [1], our finding indicates that the poor performance by patients with schizophrenia can be affected by the course of the illness. Meanwhile, it should be noted that healthy controls showed no significant correlation between scores of the Similarities subtest and rates of correct theme identification. This may be attributed to the ceiling effect by consistently high performance because our task was relatively easy for them. In other words, their relatively high correct rates and small variance (see Table 1) could mask the correlation. Therefore, the advantages of our new task may be limited to patients with difficulties in abstract thinking.

Patients showed decreased eye fixation and saccadic eye movement compared with controls during both tasks, suggesting that patients used more inactive and ineffective searching to get information from stimuli. This is consistent with a previous finding that patients had decreased eye fixation and saccades [29]. In addition, deficits in eye gaze were not more remarkable during the abstract thinking task than the concrete thinking task, that is, deficits were found for both tasks. However, the rate of correct selections in patients was lower only in the abstract thinking task. These results may have occurred because the minimal demand of visual searching is higher for abstract thinking than concrete thinking. It is noteworthy that the rate of correct theme identification was correlated with fixation count in patients, but not in controls. This finding may be due to the ceiling effect, which implies that controls had already searched the stimuli with sufficient eye fixations. In contrast, patients had not usually explored the stimuli with sufficient eye fixations, and thus, differences in eye fixations may connect to changes in abstract thinking ability.

As hypothesized, our results showed a significant correlation between abstract thinking and eye fixation in patients. The eye is the most effective sensory organ for gathering information, and eye gaze may be an important factor for cognition [30]. A person who cannot integrate information from gaze may get some troubles in abstract thinking [31,32]. Because visual searching seems to be influenced by a “top-down process” [14], patients with schizophrenia who have executive dysfunctions may have ineffective searching strategies when abstract thinking is required [33]. It is unclear what mechanism is underlying this relationship between defects in abstract thinking and abnormalities in oculomotor movement or visual searching behaviors. The frontal eye field has been postulated to represent the region of oculomotor control [34], and the dorsolateral prefrontal cortex (DLPFC) plays a role in abstract thinking [35,36]. The DLPFC has direct connections with the cortical ocular motor areas including the frontal eye field and may be involved in the decisional processes in ocular motor behavior [37]. Furthermore, patients with schizophrenia showed functional abnormalities in the frontal eye field [38] and DLPFC [39] during eye movement. Taken together, this evidence suggests that disruptions in functional circuitry between the cortical oculomotor area and the DLPFC may be a reason for both deficits in abstract thinking and eye gaze of patients with schizophrenia. Future neuroimaging studies will be needed to verify our speculation.

In terms of emotion, patients with schizophrenia showed lower rates of correct theme identification than controls, particularly during the negative emotional condition. According to previous studies, normal people showed better task performance when the emotional valence and arousal were prominent [40], while patients with schizophrenia exhibited low task performance during a negative emotional situation [41,42]. Furthermore, patients with schizophrenia also had reduced prefrontal activity while negative emotional stimuli were presented [43]. As mentioned above, the DLPFC is important to thinking abstractly. Thus, our finding of more deficits in abstract thinking during negative emotional conditions may also reflect prefrontal dysfunction.

Moreover, we found a negative correlation between abstract thinking ability and severity of social anhedonia. This is in line with a previous finding of the significant relationship between abstract thinking and negative symptoms in schizophrenia [44]. Considering that deficits in abstract thinking may make it difficult to perform social behaviors [2,45] and that problems in social behavior may be a stressful condition triggering the emergence of anhedonia [46], it makes sense that impaired abstract thinking can contribute to social anhedonia as well. On the other hand, social anhedonia may cause social dysfunctions [45], and social dysfunctions may limit the development of mental ability including abstract thinking [47]. Therefore, social anhedonia seems to have some effects on abstract thinking as well. Accordingly, we can only explain that abstract thinking is closely correlated with social anhedonia. A future longitudinal study is needed to characterize the causal relationship between abstract thinking and social anhedonia in schizophrenia.

There are some limitations in this study. Our patients were taking antipsychotic medications. Several antipsychotics can create extrapyramidal symptoms such as muscle rigidity, and they could impair eye movements in patients. It should be noted, however, that there were no significant correlations between scores of the SAS, doses of antipsychotics, and gaze results. There was a significant group difference in years of education, although intelligence was not significantly different between patients and controls. The order of the tasks was not counterbalanced across subjects to avoid the exposure to the visual stimuli before theme identification. Because the sex identification task was always performed later, the lack of group difference in the performance of sex identification might be due to the greater effect of practice and familiarity with the stimuli among patients. This study had a relatively small sample size, and it was not clear in our design whether the relationship between eye gaze and abstract thinking remained stable over time.

## Conclusions

Our results suggest that patients with schizophrenia exhibit impaired abstract thinking and decreased quality of gaze. This study provides evidence that deficits in eye gaze may be associated with impaired abstract thinking in schizophrenia. Theme identification and eye gaze tasks may be a useful tool for objective measurement of abstract thinking in patients with schizophrenia. Future studies are needed to clarify the mechanism of the link between abstract thinking and eye gaze in schizophrenia.

## Abbreviations

IAPS: International affective picture system; AOI: Area of interest; RPM: Raven’s standard progressive matrices; PANSS: Positive and negative syndrome scale; SAS: Simpson-angus scale; ANOVA: Analysis of variance.

## Competing interests

All authors declare that they have no conflicts of interest, including no financial, personal or other relationships with other people or organizations.

## Authors’ contributions

JO collected the data, analyzed and interpreted data, drafted the manuscript. JJK conceived the idea, designed the study, and participated in writing up and revising the manuscript. JWC and JSL collected the data and assisted data analysis and paper writing. All authors read and approved the final manuscript.
